# Zinc uptake promotes myoblast differentiation via Zip7 transporter and activation of Akt signalling transduction pathway

**DOI:** 10.1038/s41598-018-32067-0

**Published:** 2018-09-11

**Authors:** Hayk Mnatsakanyan, Roser Sabater i Serra, Patricia Rico, Manuel Salmerón-Sánchez

**Affiliations:** 1 Centre for Biomaterials and Tissue Engineering (CBIT) Universitat Politècnica de València, 46022 Valencia, Spain; 2 Biomedical Research Networking Centre in Bioengineering, Biomaterials and Nanomedicine (CIBER-BBN), Valencia, 46022 Spain; 3 Centre for the Cellular Microenvironment, Division of Biomedical Engineering, School of Engineering, University of Glasgow, Glasgow, G12 8LT United Kingdom

## Abstract

Myogenic regeneration occurs through a chain of events beginning with the output of satellite cells from quiescent state, formation of competent myoblasts and later fusion and differentiation into myofibres. Traditionally, growth factors are used to stimulate muscle regeneration but this involves serious off-target effects, including alterations in cell homeostasis and cancer. In this work, we have studied the use of zinc to trigger myogenic differentiation. We show that zinc promotes myoblast proliferation, differentiation and maturation of myofibres. We demonstrate that this process occurs through the PI3K/Akt pathway, via zinc stimulation of transporter Zip7. Depletion of zinc transporter Zip7 by RNA interference shows reduction of both PI3K/Akt signalling and a significant reduction of multinucleated myofibres and myotubes development. Moreover, we show that mature myofibres, obtained through stimulation with high concentrations of zinc, accumulate zinc and so we hypothesise their function as zinc reservoirs into the cell.

## Introduction

Skeletal muscle is a heterogeneous, dynamic and plastic tissue, which comprises approximately 40% of adult human body mass. Through contraction and relaxation, skeletal muscles provide movement and stability to the body. Muscle tissue contributes significantly to the correct metabolic functions serving as the major body’s reservoir of amino acids needed to maintain protein synthesis in vital tissues and organs^1^. Furthermore, muscle tissue provides storage for carbohydrates and other elements such as zinc or calcium^1,2^. Alterations in muscle mass may cause some of the most common diseases and conditions such as obesity and diabetes in addition to others chronic diseases^2^.

Muscle tissue is the largest cellular compartment of the body, characterized in physiological conditions by a relatively slow turnover^3^. It is composed by a combination of myofibres bound by connective tissue^1,4^. Satellite cells, mostly in a quiescent state and located between the basal lamina and the plasma membrane of myofibres, are the main source of myogenic precursors and provide skeletal muscle remarkable ability to regenerate after injury^5^.

In response to a muscle injury, satellite cells become activated, leave quiescence and start to proliferate. Activated satellite cells progress to become fusion-competent myoblast^6^. Eventually, these myoblasts proliferate and differentiate creating new myofibres and restoring tissue damage^7^. Various mechanisms and signalling molecules play an important role during muscle regeneration. In the first steps of post-injury, muscle degeneration and posterior inflammation result in the activation of resident macrophages, which release chemoattractant molecules recruiting neutrophils and monocytes. Subsequently, inflammatory mediators such as tumour necrosis factor alpha (TNFα) are also released. Immune, myogenic, and fibroblastic cell interactions are coordinated to eventually carry out muscle restoration^8^.

Several growth factors such as insulin-like growth factor (IGF), basic fibroblastic growth factor (bFGF), hepatocyte growth factor (HGF) or nerve growth factor (NGF) play a variety of relevant functions during muscle regeneration, enhancing damaged muscle healing. Among the signalling processes which lead to muscle regeneration, IGF/PI3K/Akt cascade is one of most studied because of its role in initial cell proliferation, myoblast commitment, and posterior differentiation and maturation to obtain new myofibrils^9–11^. Protein kinase Akt activation by IGF/PI3K cascade enhances the activity of the transcription factor MyoD in myoblasts cells, inducing them to terminal differentiation into myocytes and subsequent fusion into regenerating myofibres^12–14^.

Skeletal muscle possesses a robust innate capability for repair, however severe injuries that result in significant loss of muscle mass exceed the innate regeneration and require intervention to restore its normal function^15^. The main strategies currently under investigation to address skeletal muscle disorders and regeneration are based on drugs/biomolecules delivery, cell therapies, or a combination of both approaches. Exogenous addition of specific molecules that involve PI3K/Akt signalling pathway, such as apelin-13 peptide, Sphingosine 1-phosphate lipid (S1P)^16,17^, or growth factors, such as IGF or vascular endothelial growth factor (VEGF) have shown promising results as a potential therapeutic approach^18,19^. However, the use of growth factors has been controversial as typically involves supra-physiological doses to be effective, which increases cancer risk and other off-target lateral effects^20–22^.

In this context, cell exposure to heavy metal ions, such as Zn^+2^ and Cu^+2^ has been reported to stimulate PI3K/Akt signalling, which is known to be antiapoptotic and cytoprotective^23,24^. Zinc is one of most important transition metals present in eukaryote cells and plays a key role in the regulation and functioning of many signalling and structural proteins and transcription factors^25,26^. To achieve the control of zinc homeostasis in cells, there are several ion transporters responsible for allowing the influx of zinc from extracellular medium or different vesicles (zincosomes, Golgi apparatus or endoplasmic reticulum (ER)) to the cytoplasm. Influx zinc transporters are classified into the family of solute carriers Zip (or Slc39a) and divalent metal transporters DMT-1^27,28^. In addition of the influx transporters aforementioned, there are other transporters which drive the efflux of zinc from cytoplasm to extracellular medium and vesicles, classified into the family of Slc30a, also known as ZnT^27,28^. Among the Zip family of metal ion transport proteins, zinc transporter Zip7 (or slc39a7) is one of most studied as it is involved in signalling pathways and diseases such as cancer^29,30^. Zip7 is also the most expressed transporter among the Zip family in myoblast cells^31^. The activity of Zip7 by phosphorylation of casein kinase 2 (CK2) and posterior release of zinc from ER might be required for the activity of several protein kinases^32^.

Zinc homeostasis is of major significance in skeletal muscle tissues. Marginal zinc deficiency is manifested as muscular tissue degeneration and loss of muscle mass. It has been reported that recovery from muscle injury in mice might be partly impaired and delayed with a diet poor in zinc^33^. Furthermore, zinc can act as secondary messenger, performing signal transduction and activating some signalling pathways such as PI3K/Akt and Mek/Erk, which are highly involved in skeletal muscle regeneration^34–36^. The role of zinc in cell proliferation via PI3K/Akt and Mek/Erk pathways has been described by several authors, demonstrating the mitogenic activity of zinc^37,38^. These evidences suggest that zinc could play a key role in the regulation of muscular tissue regeneration as it is involved in several signalling pathways relevant for both myoblast proliferation and myogenic differentiation.

Here, we investigate whether exogenous zinc promotes myogenic differentiation, to assess the potential of zinc to promote muscle regeneration in the absence of growth factors. We use C2C12 murine myoblasts, a cell line widely accepted to study muscle development^39^. We investigated Akt activity as well as Zip7 expression and distribution in both undifferentiated and differentiated cells exposed to Zn^2+^ to unveil the role of zinc in myoblast differentiation.

## Materials and Methods

### Cell culture

Murine myoblasts (C2C12, Sigma-Aldrich) were cultured in high glucose Dubelcco’s Modified Eagle’s Medium (DMEM, Biowest) supplemented with 10% of Foetal bovine serum (FBS, Thermofisher) and 1% of Penicillin/Streptomycin (P/S, ThermoFisher) in a humidified atmosphere at 37 °C and 5% CO_2_. Cells were routinely passaged after reaching 70% confluence. Zinc chloride (Sigma-Aldrich) was used as source of Zn^2+^ for *in vitro* experiments.

### Live dead (cytotoxicity) and proliferation (total cell density and BrdU) assays

Cytotoxicity was tested for myoblast and myotubes. For myoblast cytotoxicity, C2C12 were seeded at low density (10.000 cells/cm^2^) in growth medium (DMEM/10% FBS/1%P/S). After 24 h allowing cell adhesion, culture medium was substituted for differentiation medium (DMEM/2% FBS/1% P/S) and supplemented with 20, 40, 60 and 80 μM of Zn^2+^. Cytotoxicity was determined using the Live/Dead Viability/Cytotoxicity Kit (ThermoFisher) after 1, 3 and 5 days of culture. Different zinc concentrations were added in each additional medium change. Medium without Zn^2+^ was used as a control (w/o Zn^2+^). As a positive control of cytotoxicity, Triton X-100 (Sigma-Aldrich) was added at 0.01% to culture medium and incubated for 10 minutes before performing the cytotoxicity assay. Cytotoxicity values were obtained by fluorescent quantification of calcein-AM (Ex 485/Em 535) with plate reader Victor III (Perkin Elmer) device. Results were represented as the ratio between number of viable cells +Zn^2+^/number of viable cells w/o Zn^2+^).

For myotube cytotoxicity, C2C12 were seeded at high density (20.000 cells/cm^2^) in growth medium (DMEM/10% FBS/1%P/S). After 24 h allowing cell adhesion, culture medium was substituted for differentiation medium (DMEM/2% FBS/1% P/S). After 6 days of culture when myotubes were formed, medium was supplemented with 20, 40, 60, 80 and 100 μM during 1, 3 and 5 days.

Note that for myoblast and myotube conditions we used differentiation medium to evaluate zinc effects in low serum conditions.

C2C12 were seeded at very low density (5.000 cells/cm^2^) for determination of total cell density, and low density (10.000 cells/cm^2^) for BrdU (proliferation) assay, previous synchronisation of cell cycle by serum starvation for 24 h. Then, culture medium was substituted for differentiation medium (DMEM/2% FBS /1% P/S) supplemented with different concentrations of Zn^2+^ (20 and 40 μM) and 10 μM of BrdU for proliferation assay. Myoblasts were fixed with 4% formaldehyde after 1, 3 and 5 days of culture for determination of total cell density, and after 1 day for BrdU assay. Total cell density was analysed after image analysis quantification (ImageJ) of total nuclei stained with Hoechst (dil: 1/7.000, Sigma-Aldrich). For BrdU proliferation analysis, results were obtained as the ratio between BrdU positive cells/total number of cells.

### Myogenic differentiation visualisation

For myoblast differentiation experiments, C2C12 cells were seeded at confluence density (20.000 cells/cm^2^) or low density (10.000 cells/cm^2^) onto polystyrene plates in culture medium. After 24 h, culture medium was changed for differentiation medium (DMEM/2% FBS/1% P/S or DMEM/1% ITS/1% P/S) supplemented with Zn^2+^ 20 and 40 μM. After 6 days of culture, cells were fixed with 4% formaldehyde and blocked with TBS/BSA 1% for 1 h at room temperature. Then cells were incubated with anti-Myosin Heavy Chain (anti-MHC, dil: 1/200, Developmental Studies Hybridoma) over night at 4 °C. After primary antibody incubation, samples were rinsed and incubated with secondary antibody anti-mouse Alexa 488 (dil: 1/500, Thermofisher). Hoechst (dil: 1/7.000, Sigma-Aldrich) was used for cells nuclei staining. Samples were mounted with 85% glycerol and imaged by a Nikon Eclipse i80 fluorescence microscope. MHC positive cells were quantified by image analysis with imageJ software, and represented as the ratio between MHC positive cells/total cell number (ratio of differentiated cells) and the ratio between multinucleated myotubes/mononucleated MHC positive cells (ratio of multinucleated myotubes).

### Analysis of intracellular Zn^2+^

C2C12 cells were seeded at low density (10,000 cells/cm^2^) and intracellular Zn^2+^ was analysed at two different time points: 24 h and after myotube formation (6 days). Intracellular amount of Zn^2+^ was determined after addition of 20 and 40 µM of Zn^2+^ in the culture medium. After cell culture, cells were washed with PBS and intracellular Zn^2+^ was labelled by means of FluoZin3-AM (2 µM) system detection (Thermofisher). Fluorescence emission was analysed by Victor III plate reader (Perkin Elmer). Cells were imaged using Nikon Eclipse i80 microscope. A parallel assay was used for Hoechst staining for total cell density quantification by image analysis with imageJ software.

For analysis of intracellular Zn^2+^ after silencing of Zip7, Zip7 silenced C2C12 cells were incubated with FluoZin3-AM (2 µM) for 40 min. and washed with PBS. After that, medium with different concentrations of zinc were added (20 and 40 µM) and fluorescence emission was measured every 40 seconds during approximately 40 minutes.

### Gene expression analysis by quantitative real time PCR

Total RNA from C2C12 cells cultured for 3 and 6 days was extracted using Quick RNA Miniprep kit (ZYMO Research) and its quantity and integrity was measured using Q3000 micro volume spectrophotometer (Quawell). RNAs were reverse transcribed using Maxima First Strand cDNA synthesis kit with thermolabile dsDNAse (Thermofisher). Real-time qPCR was carried out using the PowerUp SYBR Master Mix (Thermofisher) and 7500 Real Time fast PCR system from Applied Biosystems. The reactions were run four times (independent biological experiments). The primers used for amplification were designed based on sequences found in the GenBank database and included: MyoD1 (GeneBank M18779.1, Forward: 5′-CGC TCG TGA GGA TGA GCA T-3′, Reverse: 5′-AGC GTC TCG AAG GCC TCA T-3′), Myogenin (NM_031189.2, Forward: 5′-TGC CGT GGG CAT GTA AGG T-3′, Reverse: 5′-TGC GCA GGA TCT CCA CTT TAG-3′) and GAPDH was used as a housekeeping gene (Forward: 5′-AGG TCG GTG TGA ACG GAT TTG-3′, Reverse: 5′-TGT AGA CCA TGT AGT TGA GGT CA-3′).

The fractional cycle number at which fluorescence passed the threshold (Ct values) was used for quantification using the comparative Ct method. Sample values were normalized to the threshold value of housekeeping gene GAPDH: Δ*C*_*T*_ = *C*_*T*_ (*experiments*) − *C*_*T*_ (*GAPDH*). The Ct value of the control (condition w/o zinc) was used as a reference. ΔΔ*C*_*T*_ = Δ*C*_*T*_ (*experiments*) − Δ*C*_*T*_ (*control*). mRNA expression was calculated by the following equation: $fold\,change={2}^{-{\rm{\Delta }}{\rm{\Delta }}{C}_{T}}$.

### Zip7 expression and Akt activity

Distribution of Zip7 was analysed in non-differentiation (after 24 h) and differentiation (after 6 days) cell stages by immunofluorescence, using specific antibody against Zip7 (dil: 1/200, Santa Cruz Biotechnologies). Primary antibody was incubated over night at 4 °C. After washing, anti-goat Dye Light 488 (dil: 1/500, Thermofisher) secondary antibody was added and incubated for 1 h at room temperature. Cell cytoskeleton was labelled using Alexa Fluor 555 Phalloidin (dil: 1/100, Thermofisher) and cell nucleus with Hoechst. Cells were imaged by Nikon Eclipse i80 fluorescence microscope.

For protein expression analysis, total protein extraction was performed with RIPA buffer supplemented with protease inhibitor cocktail tablets (Roche). Proteins were separated in 12% SDS-PAGE as described previously^40^. Primary antibodies against Zip7 (dil: 1/300, Santa Cruz Biotechnologies), Akt (dil: 1/1,000; Thermofisher), phospho Akt (pAkt-serine 473) (dil: 1/700; Thermofisher) and Glyceraldehyde 3-phosphate dehydrogenase (GapDH, dil: 1/5,000; Thermofisher) were incubated over night at 4 °C. Then, membranes were washed and incubated with HRP- linked secondary antibody for chemiluminiscence band detection with ECL-Plus reactive (Thermofisher). Fujifilm Las-3000 imager device was used for protein bands visualisation.

### RNA interference (RNAi) experiments

C2C12 were seeded at confluence density (20,000 cells/cm^2^) in growth medium. After 24 h cells were transfected with MISSION esiRNA (Sigma-Aldrich) in X-tremeGENE siRNA Transfection Reagent (Roche), following manufacturer’s instructions. Cell transfection was carried out in Opti-MEM Reduced Serum medium (Thermofisher). MISSION siRNA Fluorescent Universal Negative Control 1, Cyanine 3 (NC. Sigma-Aldrich) was used as transfection control. Transfected myoblasts were cultured for 3 days with differentiation medium (DMEM/2% FBS/1% P/S). Then, myogenic differentiation was assessed by immunofluorescence of MHC.

### Statistical analysis

Each experiment was performed at least four times unless otherwise noted. Data were reported as mean ± standard deviation. To establish if obtained data followed a normal distribution, D’Agostino-Pearson omnibus test was resorted. Results were analysed by one-way ANOVA using GraphPad Prism 6.0. When differences were determined to be significant, pairwise comparisons were performed using a Tukey in case of normal distribution of data or a Dunn’s test in the opposite case. A 95% confidence level was considered significant.

## Results

### Zn^2+^ increases myoblasts proliferation

**Figure 1:**
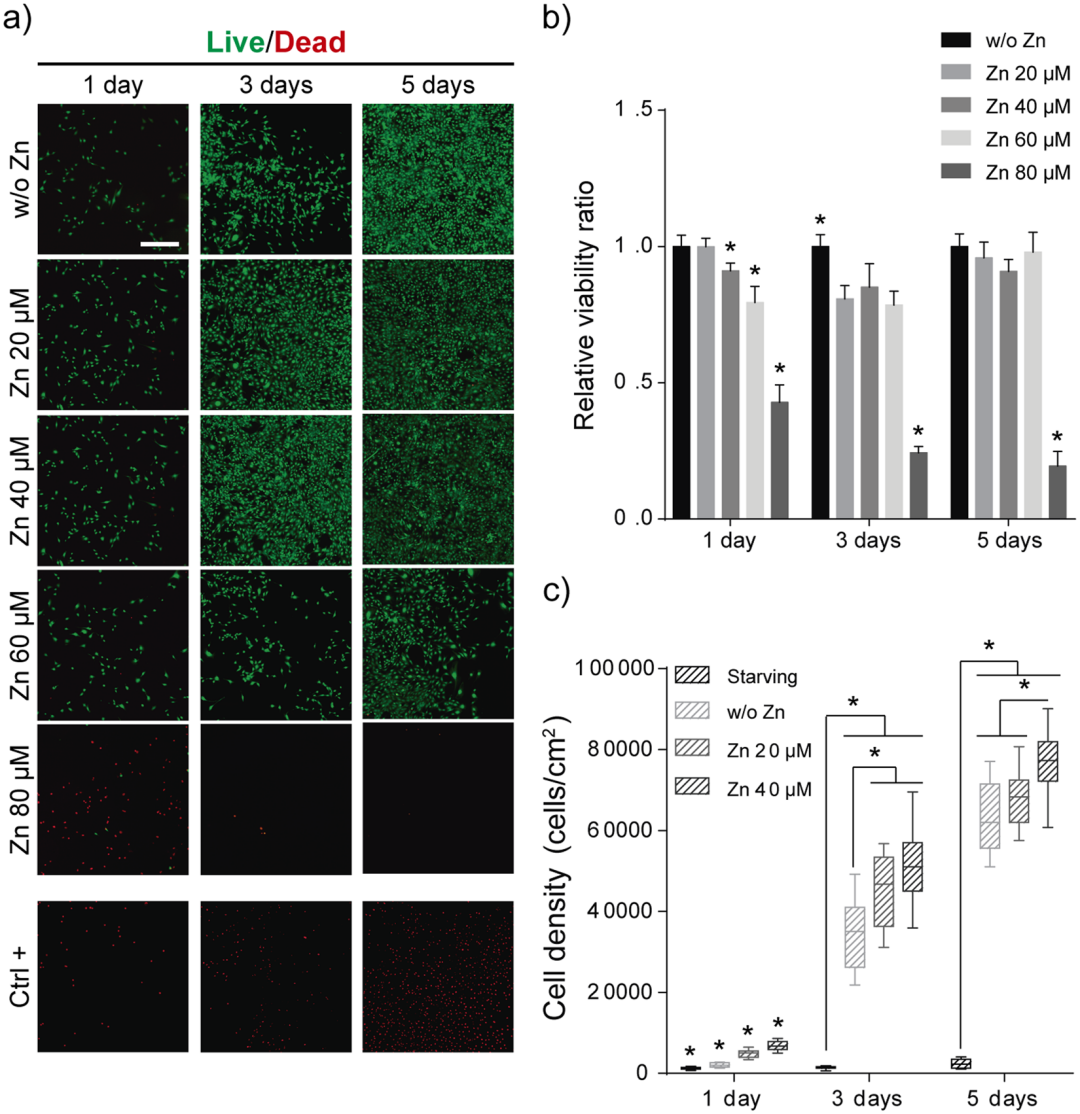
Determination of toxic concentrations of Zn^2+^ for undifferentiated muscle cells. (**a**) Live/Dead images of myoblasts (non-differentiated cells) (Scale bar: 400 μm). (**b**) Viable cells ratio (number of viable cells +Zn/number of viable cells w/o Zn) obtained by quantification of calcein-AM fluorescence signal using a plate reader. (**c**) Total cell density obtained after 1, 3 and 5 days by image analysis quantification of positive Hoechst cells/cm^2^. (N = 6 independent experiments performed). Graphs show mean ± standard deviation. Significant differences were determined by ANOVA test; *p < 0.05.

Cell viability was analysed after 1, 3 and 5 days in presence of increasing concentrations of Zn^2+^ from 20 to 80 µM in order to determine Zn^2+^ mediated toxicity on myoblasts (Fig. 1a,b). After 1, 3 and 5 days of culture, cell viability was maintained in myoblast supplemented with Zn^2+^ concentrations up to 40 µM, whereas for higher Zn^2+^ concentrations (80 µM) cell viability decreased dramatically (Fig. 1b).

For proliferation experiments, we selected only viable amounts of Zn^2+^ based in cytotoxicity results, thus we discarded 60 and 80 µM concentrations. Myoblast total cell density (total nuclei/cm^2^) was analysed after supplementing cells with 20 and 40 μM Zn^2+^. Results show that Zn^2+^ increases cell density after 1, 3 and 5 days compared with control medium (without Zn^2+^) (Fig. 1c). The zinc mitogenic effect is stronger at the initial steps of proliferation (1 day) and the trend is maintained after 3 days of culture. Nevertheless, cell proliferation is reduced at longer times (from 3 to 5 days) as the cell density approaches to confluence.

### Zn^2+^ enhances myoblast differentiation

**Figure 2:**
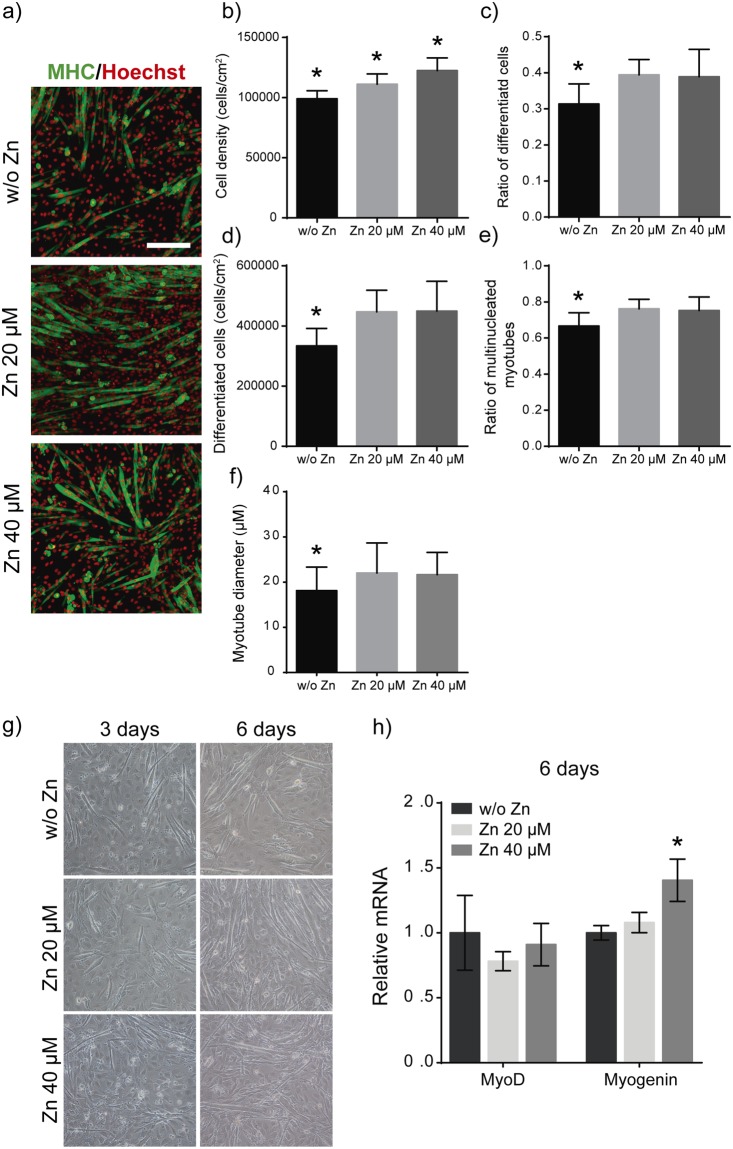
Effects of Zn^2+^ on myoblast differentiation after 6 days. (**a**) Immunofluorescence images of MHC positive staining (green) of differentiated myotubes after 6 days of culture and supplemented with different zinc concentrations. (Scale bar: 200 μm). (**b**) Quantification of total cell density after myogenic differentiation represented as the total Hoechst positive cells/cm^2^ (total nuclei). (**c**) Quantification of total differentiated cells represented as the ratio between MHC positive cells/total cell number. (**d**) Quantification of differentiated cell density represented as the total of MHC positive cells/cm^2^. (**e**) Quantification of multinucleated myotubes represented as the ratio between multinucleated myotubes/mononucleated MHC positive cells. (**f**) Myotube diameter quantification obtained after analysis of at least 30 myotubes from 5 random imaged fields. Only myotubes with 4 or more nuclei were measured (N = 5 independent experiments performed). Significant differences were determined by ANOVA test; *p < 0.05. (**g**) Bright field images of differentiated myoblasts after 3 and 6 days under different conditions. (**h**) Analysis of relative mRNA expression of MyoD and Myogenin after 6 days of culture under differentiation conditions. (N = 4 independent experiments performed). Graphs show mean ± standard deviation. Significant differences were determined by ANOVA test; *p < 0.05.

To evaluate the effect of Zn^2+^ in myoblast differentiation we quantified the expression of Myosin Heavy Chain (MHC) and the presence of myotubes, as markers of muscle differentiation, after supplementing C2C12 growing cells seeded at initial high density (20.000 cells/cm^2^) under differentiation conditions with 20 and 40 μM of Zn^2+^. Figure 2 shows C2C12 differentiation after 6 days of culture. Quantification of Fig. 2a shows that Zn^2+^ enhances C2C12 proliferation (Fig. 2b) and promotes myogenic differentiation as quantified by either the ratio between MHC positive and negative cells or the percentage of mature myotubes. (Fig. 2c–e). Indeed, myotubes show an increment in myotube diameter in the presence of Zn^2+^ (Fig. 2f). We performed the same differentiation experiment starting with low initial cell density (10,000 cells/cm^2^) (Fig. S1). The data obtained showed the same effect of Zn^2+^ in myogenic differentiation.

To further investigate the effect of Zn^2+^ on myoblast differentiation we evaluated two myogenic regulatory factors essential for muscle differentiation, MyoD and Myogenin. Real time qPCR was performed for C2C12 cells cultured in the presence of 20 and 40 μM of Zn^2+^ under differentiation conditions (20.000 cells/cm^2^) after 3 and 6 days of culture (Figs S2 and 2g,h respectively). After 3 days of culture, no relevant differences were observed in MyoD and Myogenin levels among the different conditions analysed (Fig. S2). After 6 days of culture, differentiated myotubes were observed in the presence of 20 and 40 μM of Zn^2+^ and indeed, Myogenin expression increased for 40 μM of Zn^2^ (Fig. 2g,h), although no differences were observed for MyoD expression (Fig. 2h).

### Intracellular Zn^2+^ increases in differentiated myoblasts

**Figure 3:**
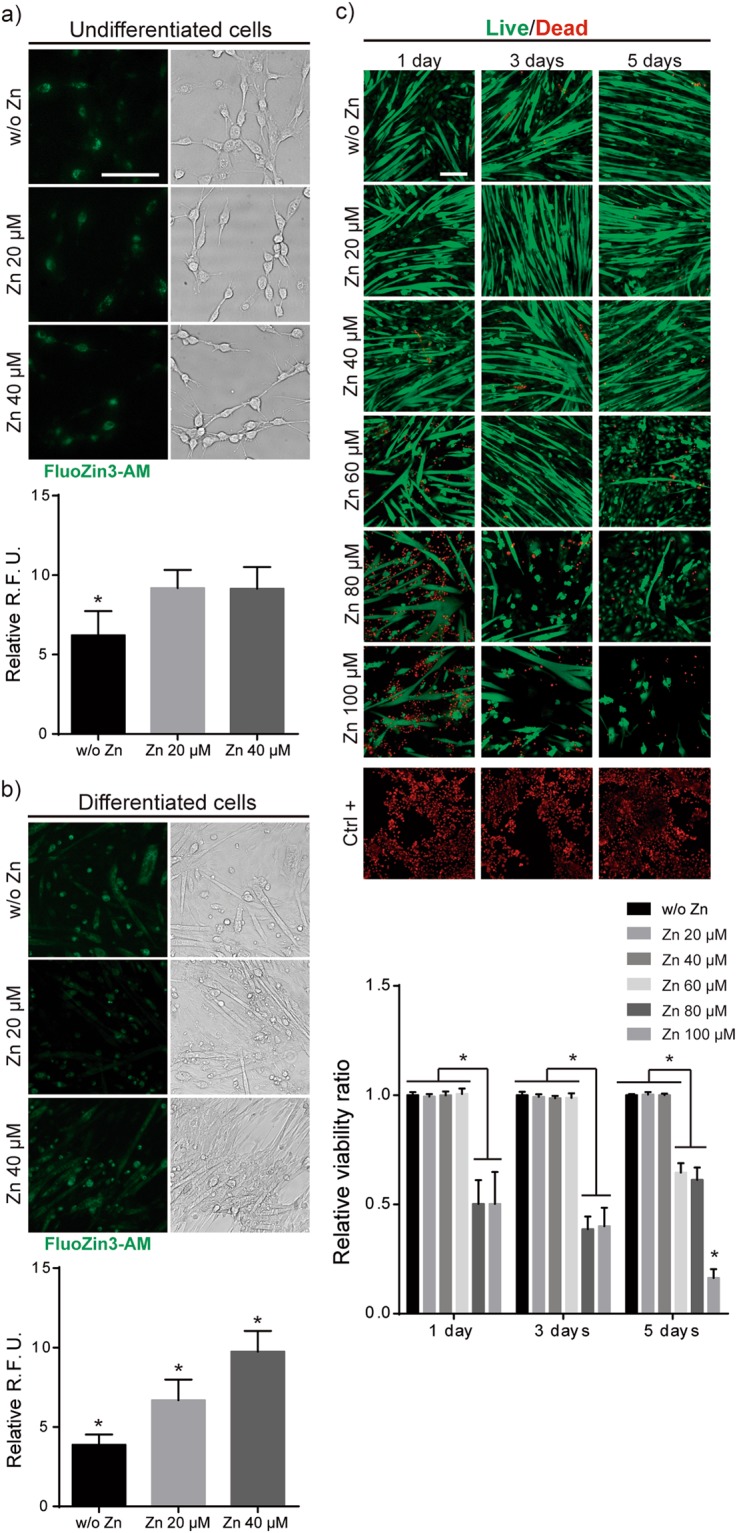
Quantification of intracellular Zn^2+^. (**a**) Bright field and fluorescence images of intracellular Zn^2+^ in undifferentiated (1 day of culture) C2C12 after Fluozin3-AM labelling. (**b**) Bright field and fluorescence images of intracellular Zn^2+^ in differentiated C2C12 (6 day of culture, myotubes) after Fluozin3-AM labelling. Values are represented as relative fluorescent units (R.F.U.) for intracellular zinc. (**c**) Live/Dead images of myotubes (differentiated cells) and quantification represented as viable cells ratio (number of viable cells +Zn/number of viable cells w/o Zn). (Scale bar: 200 μM). (N = 7 independent experiments performed). Graphs show mean ± standard deviation. Significant differences were determined by ANOVA test; *p < 0.05.

To gain insights into mechanisms induced by soluble Zn^2+^ we first measured cytosolic intake of Zn^2+^. We quantified intracellular Zn^2+^ concentration in dependence of the concentration of extracellular Zn^2+^. Free intracellular Zn^2+^ was labelled with FluoZin3-AM dye and fluorescence quantified for both undifferentiated and differentiated cells (Fig. 3a,b). Cells seeded for 24 h in presence of 20 and 40 μM of Zn^2+^ (non-differentiated stage) presented a significant increase in intracellular fluorescence emission that was not proportional to the concentration of extracellular Zn^2+^ (Fig. 3a). In contrast, for new formed myotubes after 6 days of culture, intracellular Zn^2+^ rose monotonically as extracellular concentration did (Fig. 3b). This suggests that the uptake of Zn^2+^ by differentiated myotubes is higher compared to undifferentiated myoblasts (Fig. 3a,b). Indeed, Zn^2+^ cellular distribution changes in dependence of cell stage, being localised around nuclei in undifferentiated cells and more spread throughout the cell cytoplasm in mature myotubes (Fig. 3b).

In order to clarify cell stage-dependent differences between Zn^2+^ uptakes, we next performed a new cytotoxicity assay for differentiated cells (Fig. 3c). C2C12 cells were cultured under differentiation conditions for 6 days. After obtaining new formed myotubes, their viability was measured after 1, 3 and 5 days of culture with increasing concentrations of Zn^2+^ from 20 to 100 µM (Fig. 3c). Results demonstrated that Zn^2+^concentrations up to 60 μM do not affect myotube viability, in agreement with those obtained for undifferentiated cells (Fig. 1b). However, higher cell viability (compared to undifferentiated cells) were obtained with zinc concentrations of 80 and 100 μM, which suggests that differentiated cells are more tolerant to zinc (Figs 1b and 3c).

### Expression of subcellular Zip7 transporter regulates Akt activity

**Figure 4:**
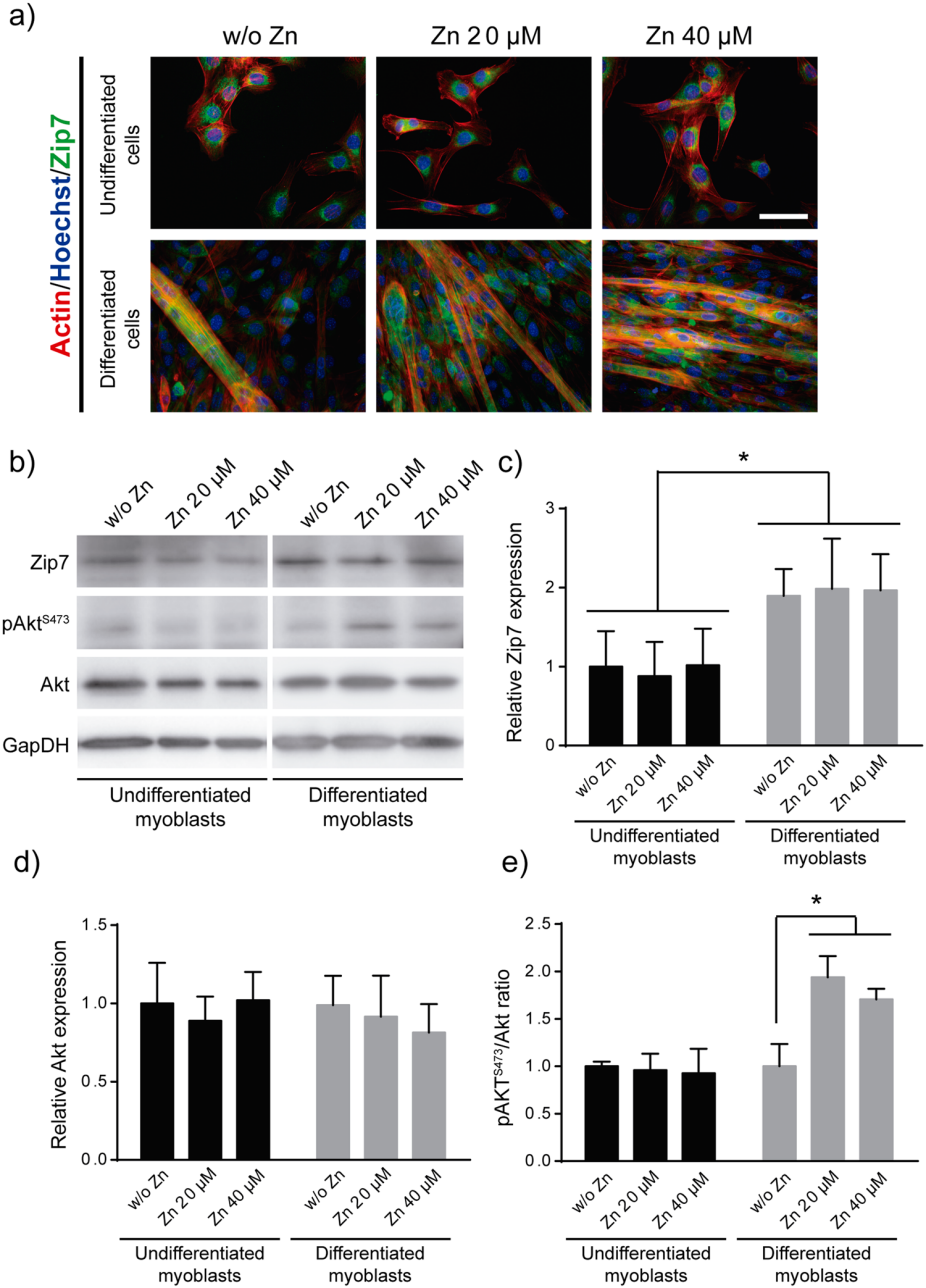
Role of Zip7 transporter and Akt activity in myoblast differentiation. (**a**) Zip7 detection by immunofluorescence (green) in undifferentiated cells (after 1 day of culture) and differentiated myotubes (after 6 days of culture). Scale bar: 50 µM. (**b**) Western blot detection of Zip7 transporter, Akt, and pAkt^S473^. GapDH was used as loading control protein. (**c**–**e**) Densitometric quantification of Zip7, Akt and pAkt^S473^/Akt ratio bands, respectively. (N = 4 independent experiments performed). Graphs show mean ± standard deviation. Significant differences were determined by ANOVA test; *p < 0.05.

Zn^2+^ transporter Zip7 has been identified as a key zinc transporter highly expressed in muscle cells^41^. It is localised in the endoplasmic reticulum (ER), Golgi apparatus and cytoplasmic vesicles^29,42^. Zip7 homodimers transport zinc into the lumen of compartments of the early secretory pathway. In order to investigate the role of Zip7 in myoblast differentiation we then analysed Zip7 protein expression and subcellular localisation in myoblast and myotubes. C2C12 cells were cultured for 1 day (undifferentiated myoblasts) and for 6 days (differentiated myotubes) under differentiation conditions. Staining for Zip7 revealed its presence around cell nuclei in undifferentiated cells (Fig. 4a, top images), resembling the distribution of intracellular Zn^2+^ observed in Fig. 3a. Nevertheless, when myoblasts fused into myotubes, Zip7 become more homogeneously distributed throughout the cell (Fig. 4a, bottom images). Zip7 western blot quantification showed a significant increase in Zip7 protein levels in differentiated myotubes (Fig. 4b,c) compared to undifferentiated cells. However, neither protein levels nor Zip7 distribution depended on the levels of free zinc in the medium, suggesting that Zn^2+^ is not directly participating in up-regulation of Zip7 expression.

In addition to the transporter function, Zip7 is able to activate several protein kinases such as Akt^24,38^. To clarify the role of zinc in cell proliferation and differentiation, we evaluated Akt signalling in undifferentiated myoblasts and mature myotubes. We examined whether zinc induced phosphorylation of Akt in serine 473 (pAkt^S473^), a requirement for full activity of Akt, by western blot. No differences were observed in total Akt expression between differentiated and undifferentiated cells (Fig. 4d). Nevertheless, Akt phosphorylation (pAkt/Akt ratio), increased in differentiated myotubes after 6 days of culture. The highest levels of pAkt were obtained when cells were supplemented with concentrations of 20 and 40 µM of zinc, which demonstrates the role of Zn^2+^ in Akt activity (Fig. 4e).

### Effect of Zip7 silencing on myoblasts

**Figure 5:**
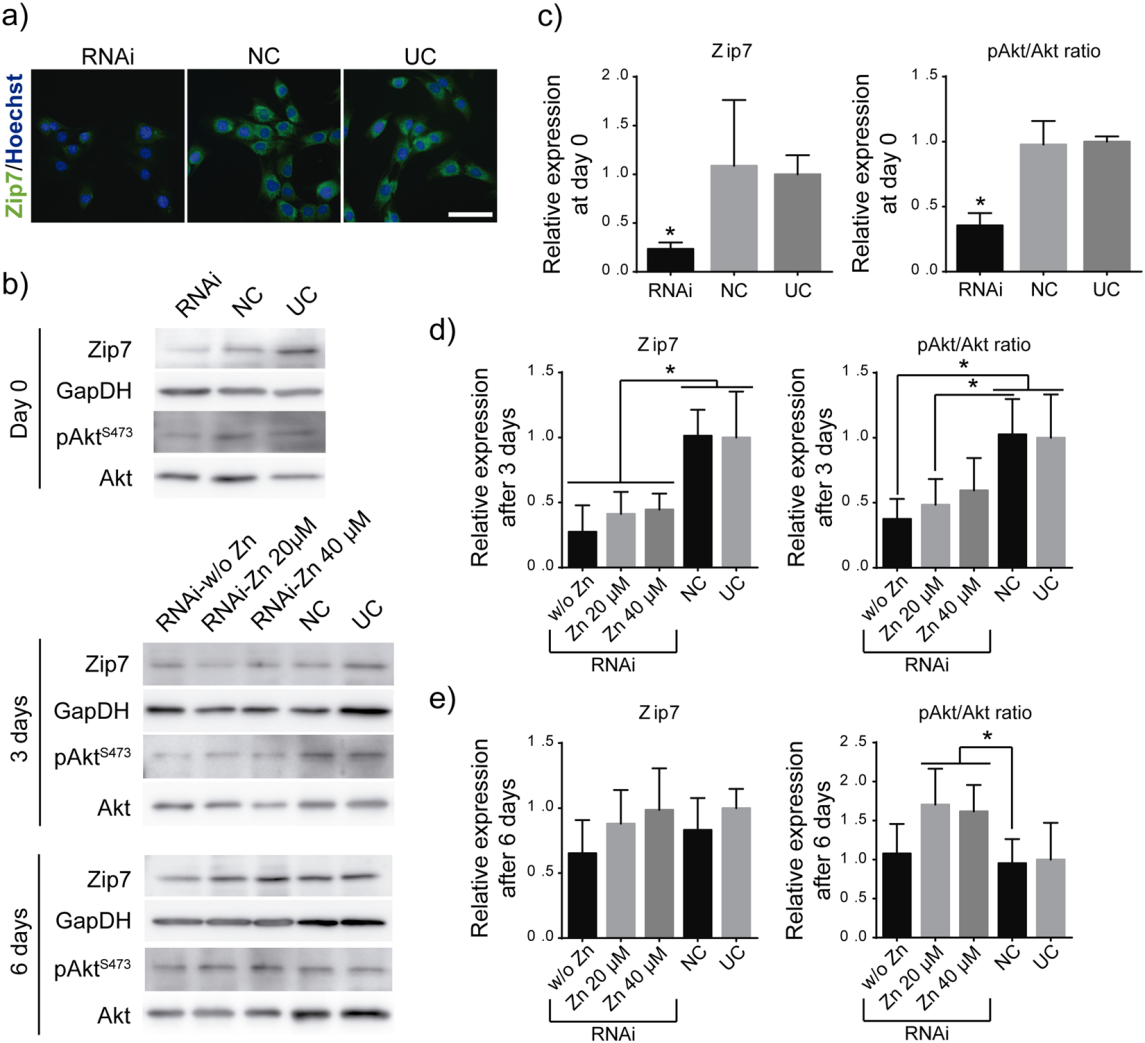
Effects of Zip7 silencing on myoblasts. (**a**) Zip7 detection by immunofluorescence (green) after mRNA silencing (Scale bar: 50 μm). (**b**) Western blot of Zip7, pAkt and Akt expression after 0, 3 and 6 days of culture. GapDH was used as loading control protein. (**c**–**e**) Densitometric quantification of Zip7 and pAkt^S473^/Akt ratio bands after 0, 3 and 6 days of culture, respectively. NC: RNAi negative control, UC: untreated cells. (N = 4 independent experiments performed). Graphs show mean ± standard deviation. Significant differences were determined by ANOVA test; *p < 0.05.

We next silenced the expression of Zip7 transporter by using RNA interference (RNAi). Transfected cells were analysed after 0, 3 and 6 days of culture under differentiation conditions. Zip7 levels were assessed by immunofluorescence (Fig. 5a) and western blot (Fig. 5b–e). Myoblast knocked down by RNAi showed the lowest levels of Zip7 immunostaining compared to RNAi negative control (NC, transfected with scrambled siRNAs) and untreated cells (UC, non-transfected cells) (Fig. 5a). Western blot experiments revealed reduced levels on Zip7 expression (23.5%) on cells transfected with siRNA against Zip7 after 1 day of culture compared with untreated cells (Fig. 5b,c). After 3 days of culture, the levels of Zip7 protein expression increased until 27.5%, and after 6 days Zip7 levels were restored to the original ones (Fig. 5b,d). This was expected due to the transitory effect of this RNAi silencing. Addition of 20 and 40 µM of Zn^2+^ did not result in any significant effect on Zip7 silencing at any time point. However, knocking down Zip7 altered Akt activity. pAkt^S473^/Akt ratio diminished immediately after RNAi treatment, (control at day 0, Fig. 5b,c) progressively raised after 3 days (Fig. 5d) and was fully restored after 6 days (Fig. 5e). After 6 days of culture, and we note that after this time Zip7 silencing was not effective, we observed again that Zn^2+^ presence increased the level of Akt phosphorylation (pAkt/Akt ratio).

### Silencing of Zip7 alters intracellular Zn^2+^ content

**Figure 6:**
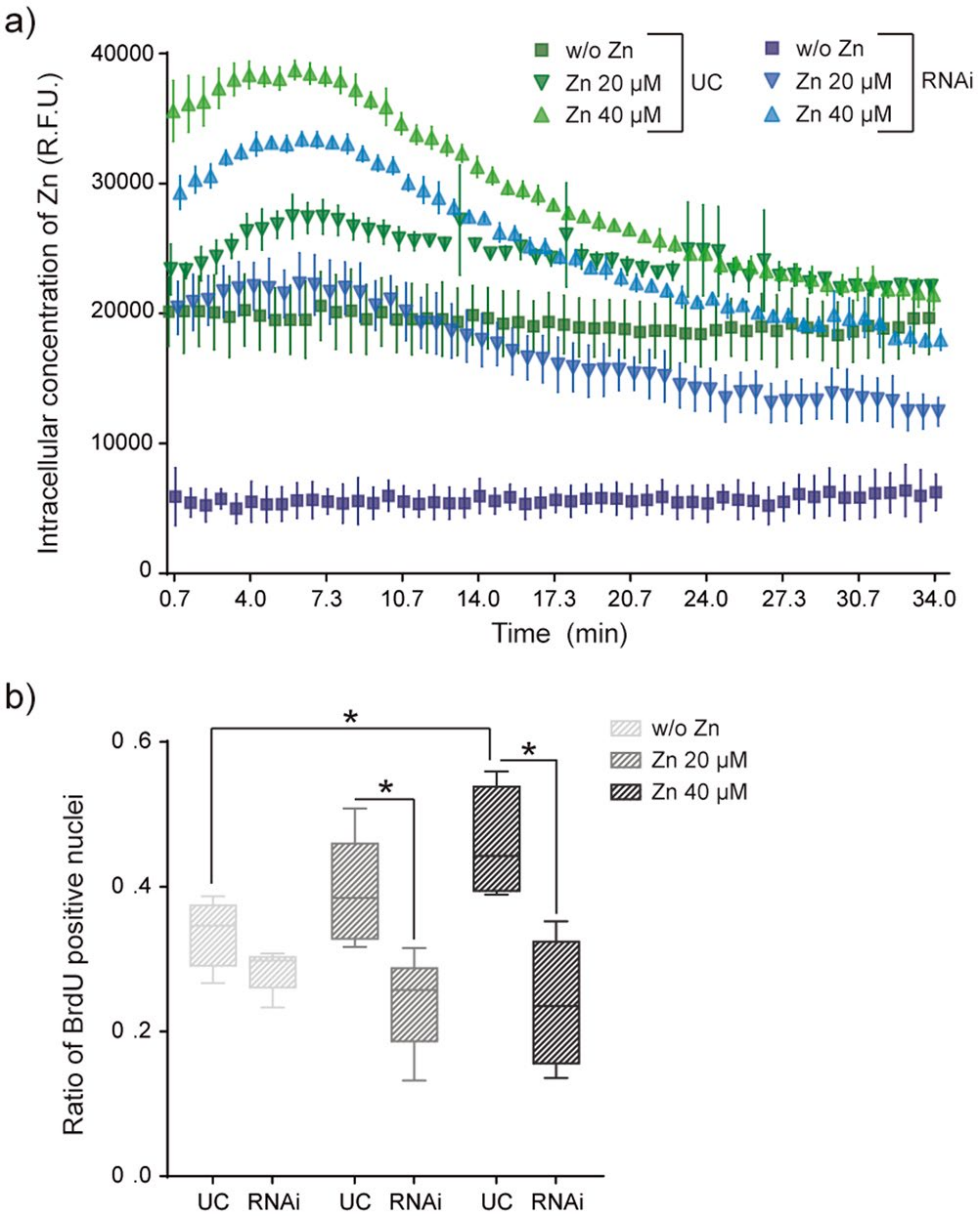
Effects of Zip7 silencing in intracellular Zn^2+^ homeostasis and proliferation. (**a**) Intracellular Zn^2+^ measurements after Fluozin3-AM labelling in control (UC, untreated cells) and Zip7-deficient cells. Fluorescence emission was measured every 40 seconds. Values are represented as relative fluorescent units (R.F.U.). (N = 6 independent experiments performed). (**b**) Myoblasts proliferation was determined by BrdU labelling followed by quantification of immunofluorescence. Values are represented as the ratio between BrdU positive cells/total nuclei (N = 5 independent experiments performed). UC: untreated cells. Graphs show mean ± standard deviation. Significant differences were determined by ANOVA test; *p < 0.05.

To further investigate the role of Zip7 in intracellular zinc homeostasis, we next quantified intracellular Zn^2+^ concentration after Zip7 silencing. We labelled free intracellular Zn^2+^ with FluoZin3-AM dye. After that, cells were supplemented with Zn^2+^ 20 and 40 µM and fluorescence emission was quantified every 40 seconds. Figure 6 shows that immediately after addition of zinc-supplemented medium to untreated cells (UC in Fig. 6), intracellular concentration of Zn^2+^ increased significantly compared to control condition w/o Zn^2+^ (Fig. 6a-green labels). Nevertheless, the measured values of intracellular Zn^2+^ obtained in Zip7-deficient cells were lower in all conditions compared to the equivalent UC (Fig. 6a-blue labels). Despite intracellular zinc concentration rapidly increased after zinc addition, the values progressively decreased until stabilisation, and this effect was more pronounced in Zip7-silenced cells. This observation together with the fact that the basal amount of intracellular zinc (condition w/o zinc) is strongly reduced in Zip7-deficient cells, confirm the role of Zip7 in intracellular zinc homeostasis. Note that the basal amount of intracellular zinc in control condition (w/o Zn) can be only originated by zinc-released from intracellular organelles.

In addition, cell proliferation was also affected after Zip7 silencing. We evaluated Zn^2+^-dependent cell proliferation in Zip7-deficient cells by BrdU assay. Figure 6b shows that BrdU positive cells rise monotonically as extracellular concentration of zinc does in control condition, whereas this effect was reverted after Zip7 silencing with no differences in BrdU levels regardless of the concentration of Zn^2+^ in the culture medium (for images see Fig. S3).

### Effects of Zip7 silencing on myotubes

**Figure 7:**
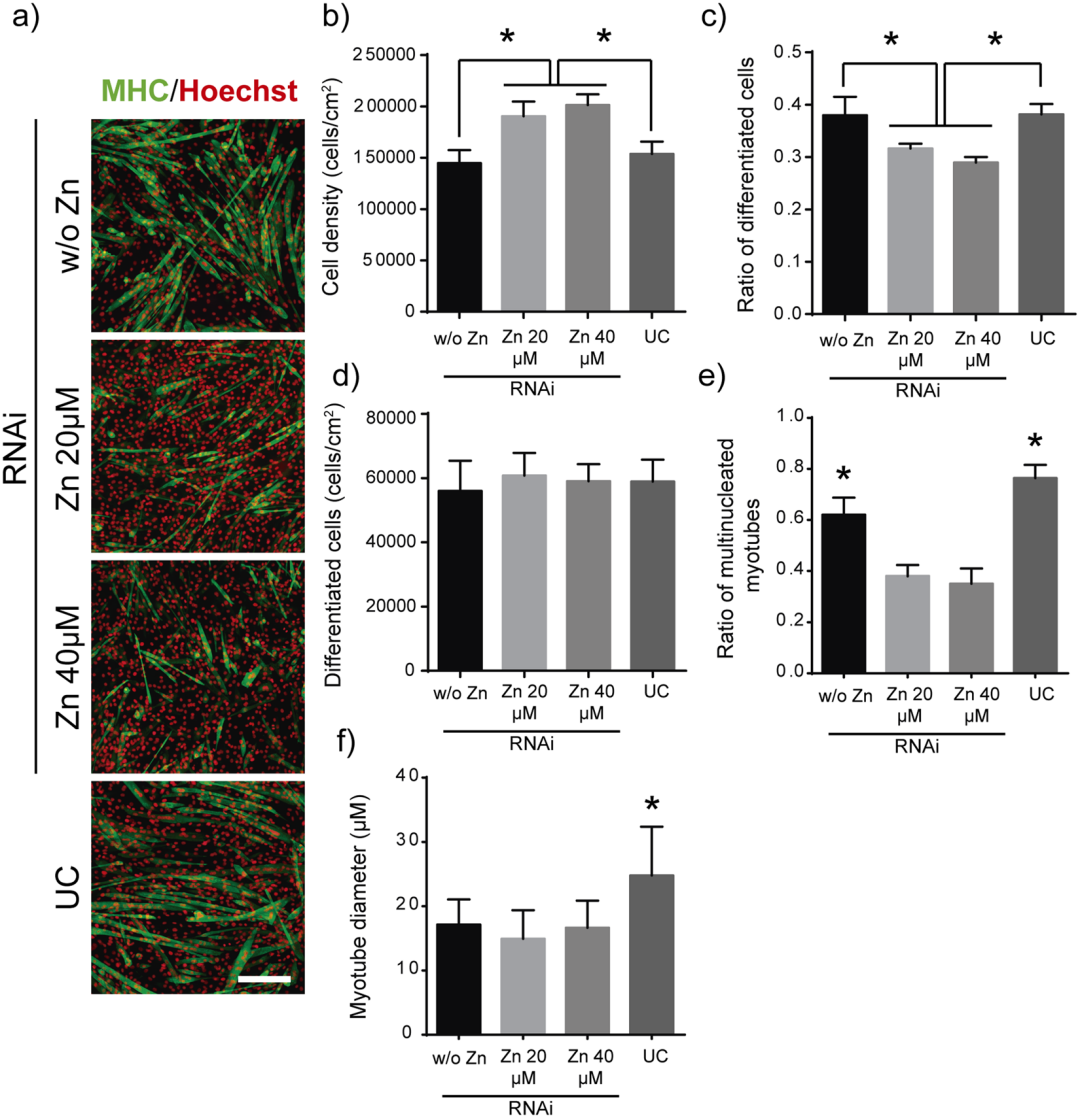
Effects of Zip7 silencing in myotubes after 6 days of culture. (**a**) Immunofluorescence images of MHC positive staining (green) of differentiated Zip7-deficient myotubes after 6 days of culture and supplemented with different zinc concentrations. (Scale bar: 200 μm). (**b**) Quantification of total cell density after myogenic differentiation represented as the total Hoechst positive cells/cm^2^ (total nuclei). (**c**) Quantification of total differentiated cells represented as the ratio between MHC positive cells/total cell number. (**d**) Quantification of differentiated cell density represented as the total of MHC positive cells/cm^2^. (**e**) Quantification of multinucleated myotubes represented as the ratio between multinucleated myotubes/mononucleated MHC positive cells. (**f**) Myotube diameter quantification obtained after analysis of at least 30 myotubes from 5 random imaged fields. Only myotubes with 4 or more nuclei were measured. Graphs show mean ± standard deviation. (N = 5 independent experiments performed). Significant differences were determined by ANOVA test; *p < 0.05.

We next evaluated the effects of blocking Zip7 protein expression on myotube formation. Zip7 knockdown had no effect either on total cell density or on ratio of total differentiated cells (control condition w/o Zn^2+^) expressing MHC compared to untreated cells (Fig. 7a–c). However, in cell cultures supplemented with 20 and 40 µM of Zn^2+^, blocking of Zip7 protein expression resulted in a significant reduction of the ratio of differentiated cells expressing MHC despite the slight increase in cell density (Fig. 7a–c). Quantification of myotubes formed after Zip7 silencing resulted in a considerably reduction of the ratio of multinucleated myotubes formed in presence of Zn^2+^, although the total MHC positive cells remained similar in all conditions (Fig. 7d,e). In addition, myotube diameters diminished up to 31% in Zip7-deficient cells (Fig. 7f).

## Discussion

Zinc is one of the essential trace elements, ubiquitous in cell metabolism and essential to carry out eukaryote cells biological processes^25,43^. Zn^2+^ exhibits anti-apoptotic properties and enhances cell survival and proliferation^44,45^, however Zn^2+^ overload results toxic for all cells. High levels of free Zn^2+^ in the cytoplasm can induce both cell necrosis and apoptosis^46,47^. Toxic concentration of Zn^2+^ varies for different cell-types^48,49^. We have determined that 60 µM Zn^2+^ concentration is toxic for myoblasts affecting viability at early culture times (Fig. 1), while 80 µM concentration is detrimental for differentiated myotubes. Sub-toxic concentrations of Zn^2+^ (Zn^2+^-concentrations 20 and 40 µM) increased cell proliferation monotonically (Figs 1b and S1b), confirming the mitogenic effects of Zn^2+^ in C2C12^37^.

Myoblast proliferation and differentiation are two closely linked events involved in skeletal muscle regeneration. Cell proliferation allows cells to reach confluence and triggers the exit of the cell cycle^50,51^ which in turn initiates myoblast differentiation^52^. Our results regarding zinc mitogenic effects are in agreement with those previously reported by Ohashi *et al*.^37^. However, we have found opposite results in regards to myoblast differentiation. Note that in all the experiments, we have cultured cells under low serum (2% FBS, differentiation media that also allowed cell proliferation), standardised as a more relevant approach to mimic the physiological environment surrounding myoblasts after muscle injury, where proliferation precede differentiation^52^. In Ohashi’s work, they used insulin-transferrin-selenium (ITS 1%) as differentiation medium, in order to minimally supplement the media in absence of serum. Absence of serum provokes arrest of cell proliferation and promotes cell differentiation. Note that for the sake of comparison with previous published work we have also reproduced Ohashi’s experiments using ITS 1% instead of 2% FBS (Fig. S6) Then, we obtained similar results: zinc supplementation affected neither myoblast proliferation nor differentiation in serum-free media conditions^37^. The different culture media used in both cases explains the differences obtained.

It is important to note that although in our results cell density is dependent of the concentration of zinc presence in the medium (Fig. 1), this monotonic increase in cell proliferation is not directly related to cell differentiation which occurred at similar levels after the addition of 20 or 40 µM of Zn^2+^. Addition of Zn^2+^ induced myotube maturation, with more and thicker multinucleated myotubes (Figs 2a–f and S1).

We have analysed the effects of Zn^2+^ in the gene expression of MyoD and Myogenin, two essential transcription factors of myogenesis, that are differentially expressed/repressed in dependence of the cell-specific differentiated stage^53^. Results suggest that MyoD expression, which is associated with proliferating myoblasts^54^, was not dependent on the presence of Zn^2+^ after 3 or 6 days of culture (Figs S2 and 2h). However, Myogenin, which is involved in myotube maturation^54^, significantly increased after 6 days of culture in the presence of 40 µM of Zn^2+^, along with increased myotube diameter (Fig. 2f), suggesting the role of zinc in the expression of the terminal muscle phenotype rather than in early commitment of cells to myogenic lineages.

Intracellular Zn^2+^ balance is involved in many biological activities^43,47,48,55^. Intracellular zinc measurements in undifferentiated myoblasts were not correlated with the concentration of Zn^2+^ in the medium. However, after differentiation, myotubes increased the intracellular Zn^2+^ intake monotonically, and proportional to the extracellular Zn^2+^ concentration, that distributed homogeneously throughout mature myotubes (Fig. 3). These results suggest that, similarly as calcium storage within the sarcoplasmic reticulum (SR)^56^, differentiation of myoblasts and subsequent formation of SR involve Zn^2+^ storage, an essential element for endoplasmic reticulum function and protein folding^57,58^. This storage function of the SR is correlated with the fact that myotubes are viable in environments with higher extracellular Zn^2+^ concentrations, as high as 100 μM for 3 days, than undifferentiated cells (Fig. 3).

Zinc transporter Zip7 localised within the endoplasmic reticulum in undifferentiated cells but its location changes after myoblast differentiation, being homogeneously distributed, and more expressed, throughout the sarcoplasmic reticulum in differentiated cells, following the pattern of intracellular zinc (Fig. 4a). After Zip7 knock down myoblasts exhibit altered Zn^2+^ homeostasis, with lower intake of extracellular zinc and minimal release from cytoplasmic organelles (Fig. 6a), demonstrating that Zip7 plays a key role in intracellular zinc regulation. Zip7-deficient cells also presented reduced proliferation rates (Figs 6b and S3) confirming that proliferative effect of zinc is dependent of Zip7 activity.

Moreover, Zip7-deficient myoblasts presented a reduction in the percentage of differentiated cells in Zn^2+^-treated cells (Fig. 7c), as well as in the ratio of multinucleated cells and myotube diameter (Fig. 7e). Altogether, these results point out the crucial role of Zip7 protein in Zn^2+^-mediated induction of myoblast differentiation and myotube maturation, in agreement with qPCR results obtained for Myogenin expression for 40 µM zinc (Fig. 2h). The importance of Zip7 has been recently shown in *Drosophila*. Negative mutation in *Drosophila* catsup gene, mammalian Zip7 orthologous gene, causes Notch abnormal accumulation in endoplasmic reticulum and Golgi apparatus, promoting self-renewal, and inhibiting myogenic differentiation^57,59^.

**Figure 8:**
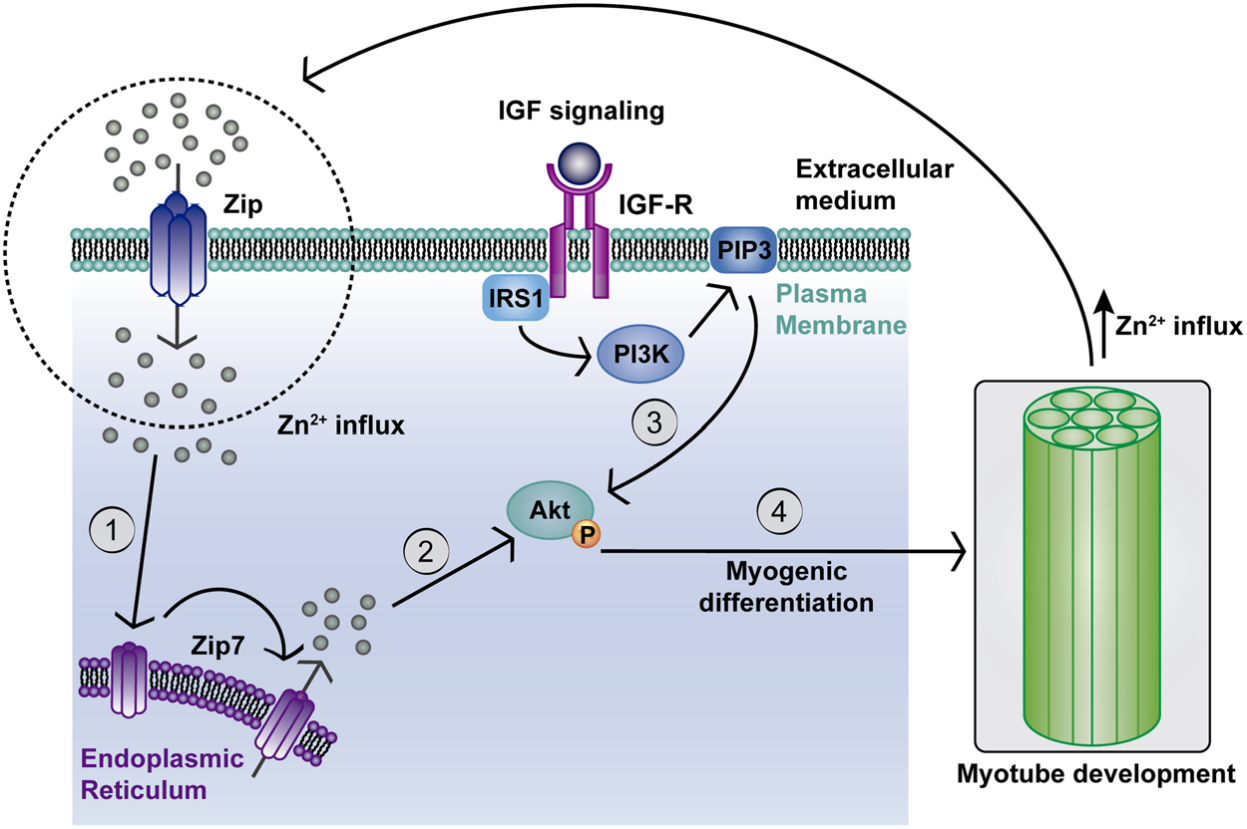
Scheme of cascade of events representing the role of zinc in the regulatory crosstalk promoting myogenesis. Zinc ions influx from extracellular medium through membrane Zip transporter mediate phosphorylation of Zip7 endoplasmic reticulum transporter. Activation of Zip7 produces a release of intracellular storage of Zn^2+^ and subsequent phosphorylation of protein kinase Akt and consequently enhances myogenic differentiation. Myotube formation, in turn, stimulate Zn^2+^ extracellular uptake, enhancing myogenic differentiation process and myotubes development. (1) References^28,35^. (2) Reference^35^. (3) References^9,37^. (4) References^9,37^.

Both *in vitro* and *in vivo* studies have shown that Akt activity, which regulate many processes including cell proliferation, survival and metabolism, is critical for optimal muscle growth and regeneration^60^. The protein kinase Akt is involved in myoblast proliferation and differentiation^10,61,62^ and is essential in earliest stages of myogenic differentiation^13^. We show that increased extracellular Zn^2+^ levels, below toxic concentration, induces an over proliferation of myoblasts and enhances cell differentiation and myotubes development. It has been reported the important role of zinc ions in Akt phosphorylation via Zip7 tyrosine kinase activator activity^29^, in a similar way to IGF/PI3K/Akt cascade^34,37^. Figure 8 depicts the chain of events leading to regulatory crosstalk between zinc and myoblasts. Zinc ions influx from the extracellular medium through Zip membrane transporters. Zn^2+^ activates phosphorylation of Zip7, which in turn increases cytoplasmic levels of Zn^2+^ promoting the phosphorylation of Akt. The phosphorylation of these proteins kinase activates mitogenic molecular pathways, enhancing myoblast proliferation^37,61,63^. We did not find any significant differences in Akt phosphorylation after 1 day of culture in the presence of extracellular Zn^2+^ for undifferentiated cells (Fig. 4d), whereas pAkt increased after 6 days for differentiated cells in Zn^2+^-treated myoblasts (Fig. 4e). These findings suggest that exogenous Zn^2+^ triggers Akt activation, promoting cell differentiation and myotube maturation as shown in the scheme in Fig. 8.

Myogenic differentiation assessed with Zip7-deficient myotubes (Fig. 7) shows that exogenous Zn^2+^ hinders myogenic differentiation at the expense of increased cell proliferation. Cell density in Zip7-deficient myotubes was found to be 32–39% higher in Zn^2+^-treated cells compared to non-treated and non-transfected cells (Fig. 7b). Moreover, Zip7 knockdown resulted in reduced Akt phosphorylation up to 3 days (Fig. 5d), suggesting that the rise of cell proliferation by Zn^2+^ action is not directly related with Akt activity. This agrees with several studies where cell proliferation was found to be related to Mek/Erk activation by Zn^2+^ ^29^^,^^34^^,^^37^. Our results demonstrate that the enhancement of myotube maturation and development induced by exogenous Zn^2+^ is closely related to Zip7 transporter and its downstreams Akt and Myogenin.

## Electronic supplementary material

Supplementary information

## Data Availability

The datasets generated during and/or analysed during the current study are available in the University of Glasgow Repository, http://researchdata.gla.ac.uk/.
