# Supplementary material for: Zinc uptake promotes myoblast differentiation via Zip7 transporter and activation of Akt signalling transduction pathway
Source: Sci Rep. 2018 Sep 11;8:13642. doi: 10.1038/s41598-018-32067-0 (PMC6133932; doi:10.1038/s41598-018-32067-0)
Supplement: Supplementary file 1 — Supplementary information [file 41598_2018_32067_MOESM1_ESM.docx]

**Zinc uptake promotes myoblast differentiation via Zip7 transporter and activation of Akt signalling transduction pathway**

Hayk Mnatsakanyan^1^, Roser Sabater i Serra^1,2^, Patricia Rico^1,2^*, Manuel Salmerón-Sánchez^1,2,3*^

^1^ Centre for Biomaterials and Tissue Engineering (CBIT) Universitat Politècnica de València, 46022 Valencia, Spain.

^2^ Biomedical Research Networking Centre in Bioengineering, Biomaterials and Nanomedicine (CIBER-BBN), Valencia, 46022, Spain.

^3^ Centre for the Cellular Microenvironment. Division of Biomedical Engineering, School of Engineering, University of Glasgow, Glasgow G12 8LT, United Kingdom.


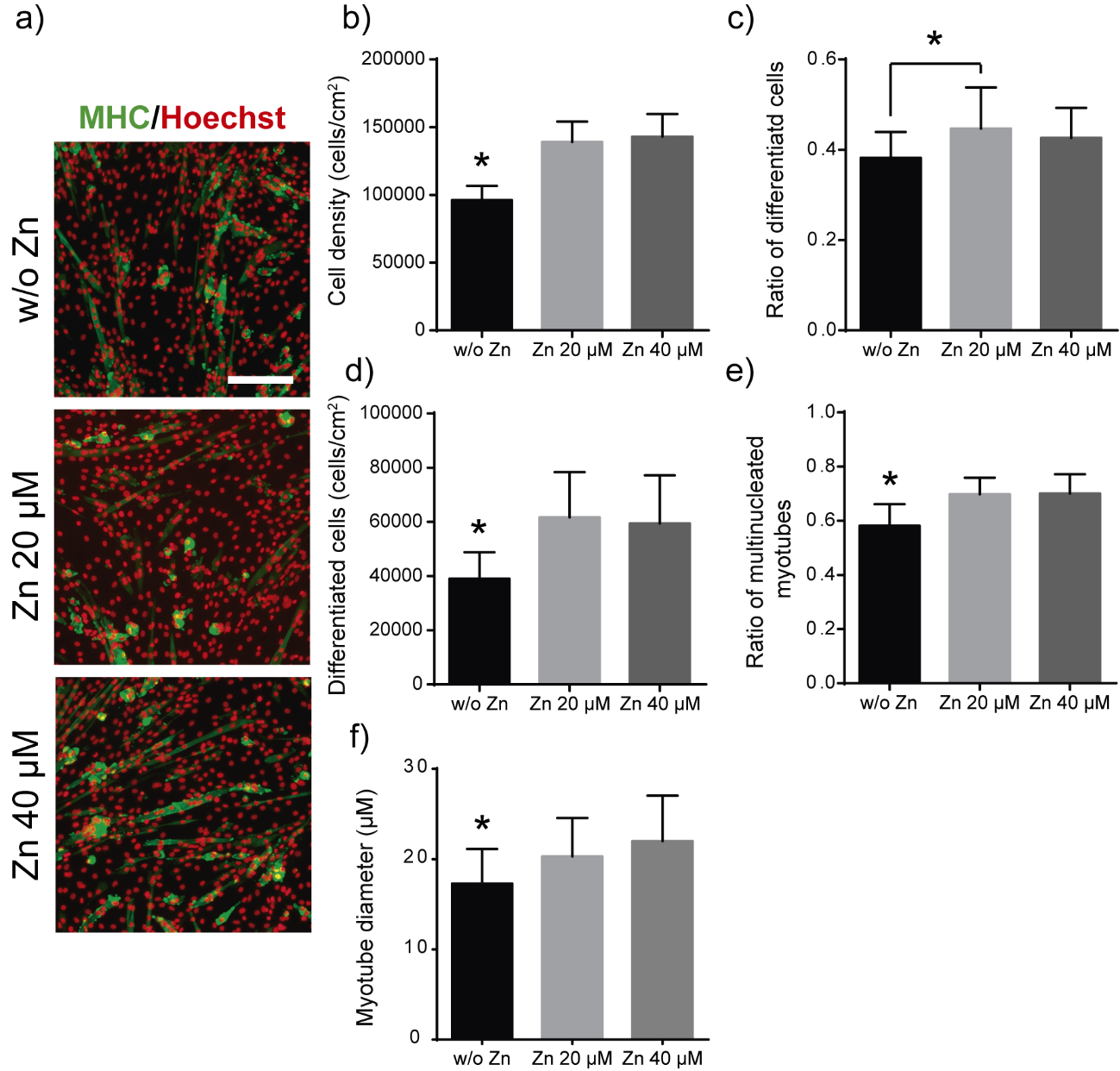


**Figure S1**. Influence of Zn^2+^ on myoblast differentiation with low initial cell density (10.000 cells/cm^2^).

a) Immunofluorescence images of MHC immunostaining (green) of differentiated myotubes after 6 days of culture and supplemented with different zinc concentrations (Scale bar: 200 μm). b) Quantification of total cell density after myogenic differentiation represented as the total Hoechst positive cells/cm^2^ (total nuclei). c) Quantification of total differentiated cells represented as the ratio between MHC positive cells / total cell number. d) Quantification of differentiated cell density represented as the total of MHC positive cells/cm^2^. e) Quantification of multinucleated myotubes represented as the ratio between multinucleated myotubes / mononucleated MHC positive cells. f) Myotube diameter quantification obtained after analysis of at least 30 myotubes from 5 random imaged fields. (N = 5 independent experiments performed). Graphs show mean ± standard deviation. Significant differences were determined by ANOVA test; *p < 0.05.


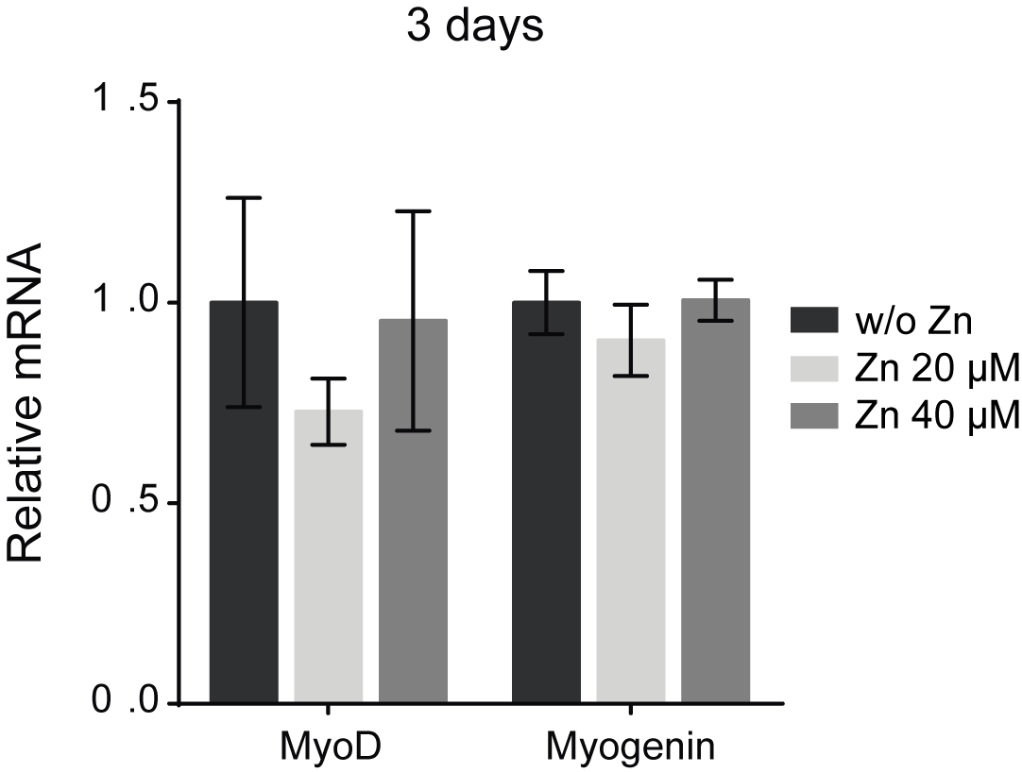


**Figure S2.** Analysis of relative mRNA expression of MyoD and Myogenin after 3 days of culture under differentiation conditions. (N = 4 independent experiments performed). Graphs show mean ± standard deviation. Significant differences were determined by ANOVA test; *p < 0.05.


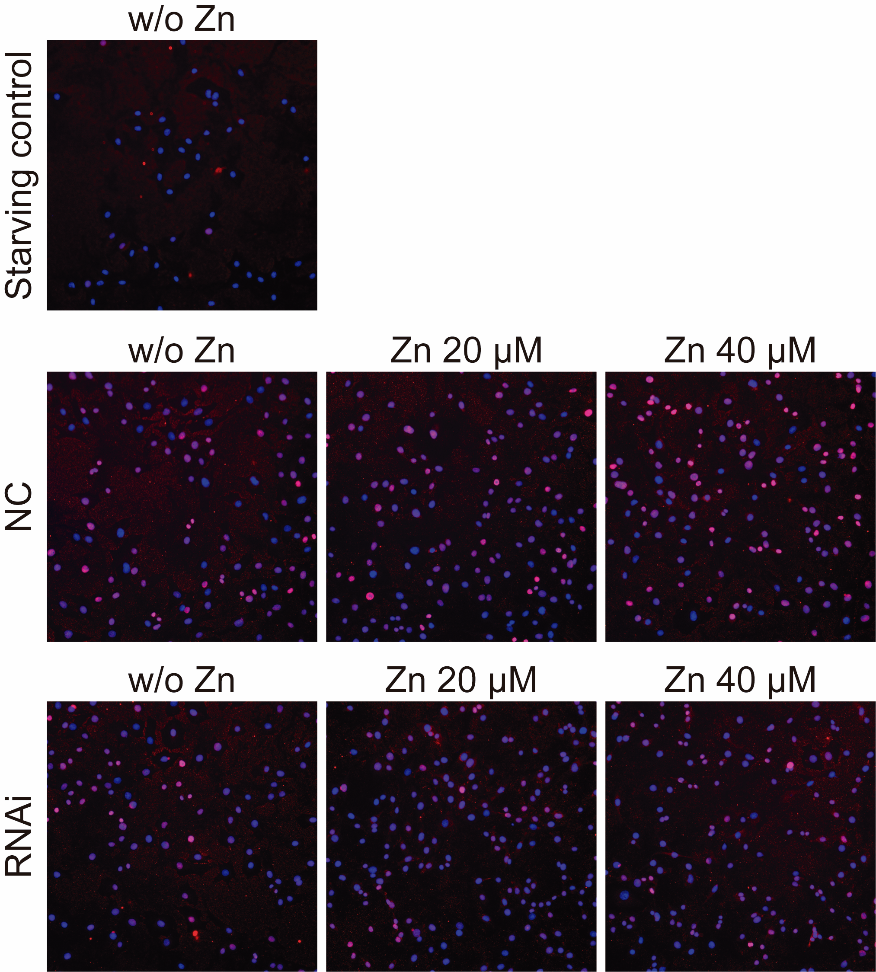


**Figure S3.** Analysis of Zinc-dependent proliferation after Zip7 silencing. Representative images of immunostaining of BrdU assay indicating proliferating cells (red) and nuclei (blue) after 24h of culture. (N = 5 independent experiments performed).


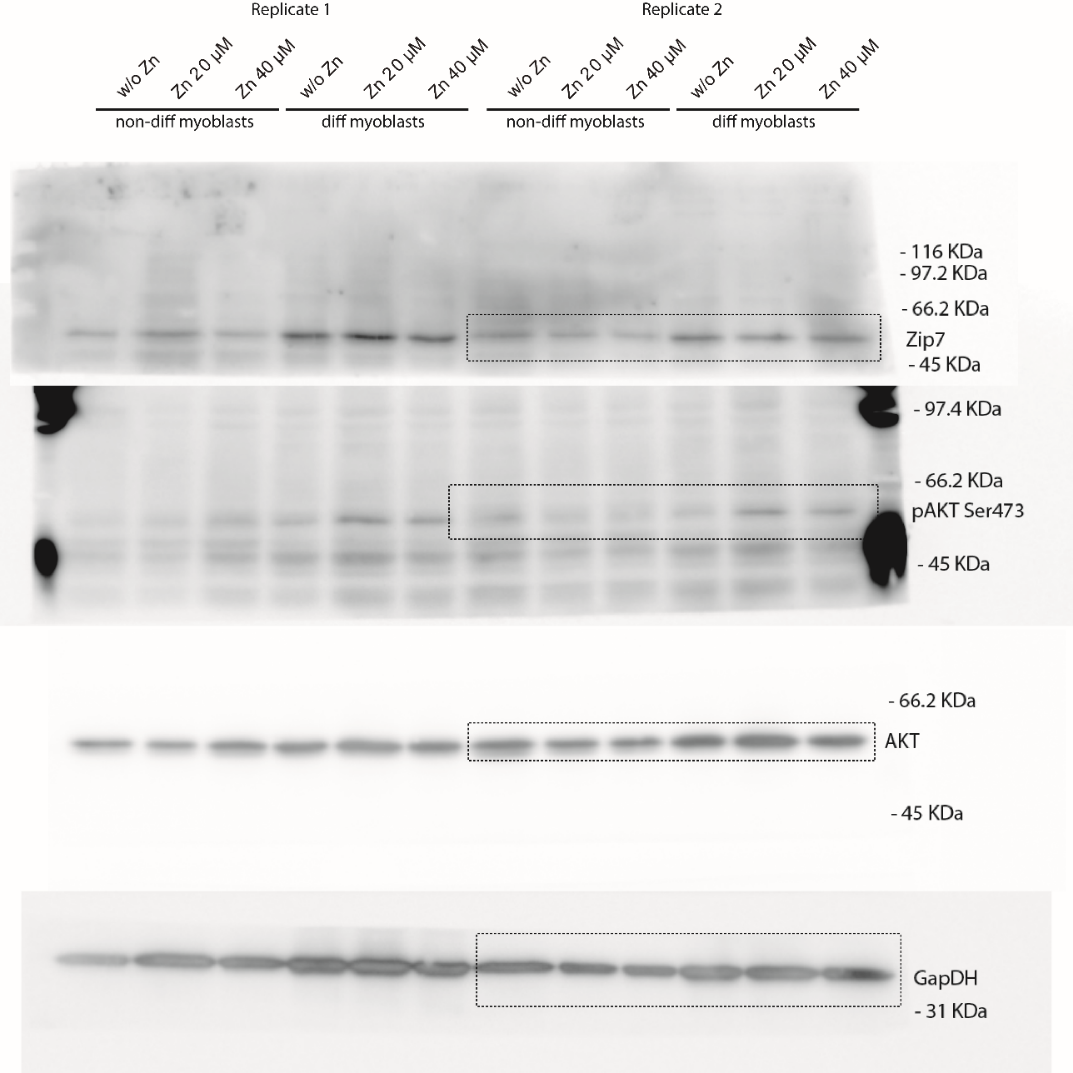


**Figure S4.** Representative immunoblots corresponding with western blot bands of figure 4. The dot grid represents the chosen pool of bands used for the corresponding figure. In each blot were run a couple of biological replicates (N= 4).


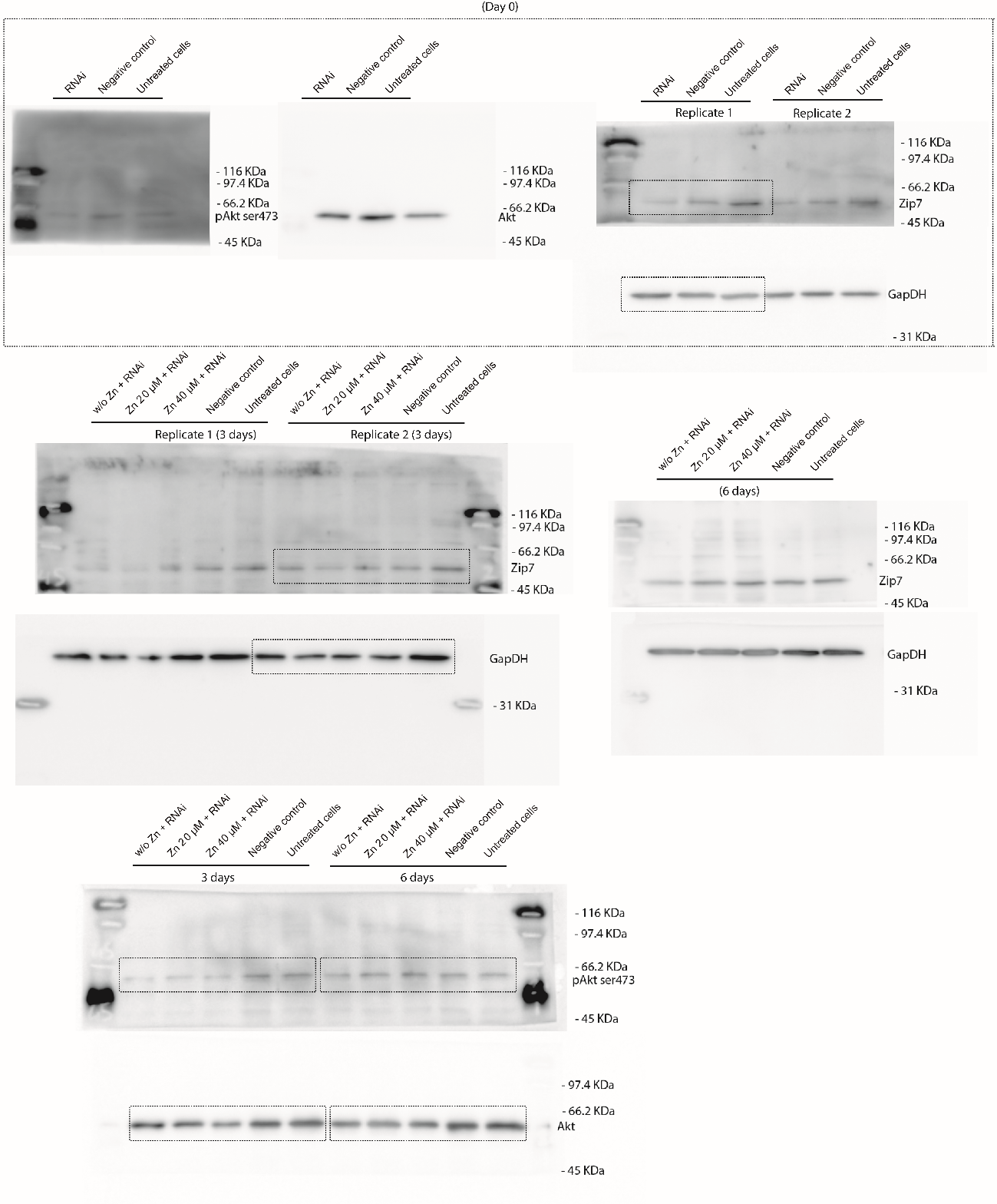


**Figure S5.** Representative immunoblots corresponding with western blot bands of figure 5. The dot grid represents the chosen pool of bands used for the corresponding figure. From up to dawn were represented the protein levels of Zip7 and the ratio of phosphorylated AKT in serine 473 in a differentiation culture of C2C12 cells (at day 0, after 3 days and 6 days) after to be treated with RNAi.


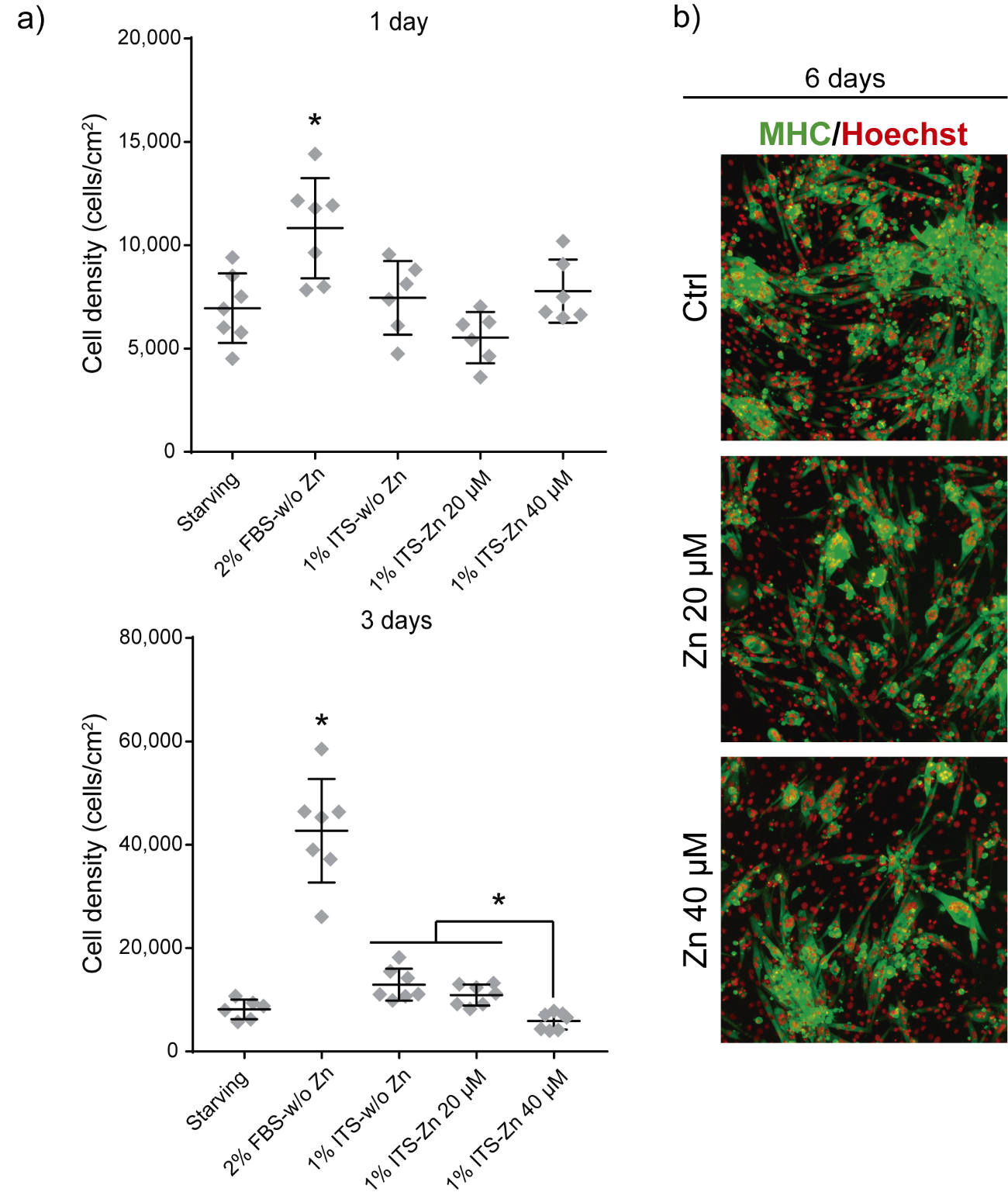


**Figure S6.** Analysis of myoblasts proliferation and differentiation using serum-free medium supplemented with 1% ITS.

a) Total cell density obtained after 1 and 3 days of culture determined by image analysis quantification of positive Hoechst cells / cm^2^. (N = 7 independent experiments performed). 2% FBS medium was used as a control for proliferating cells condition. Graphs show mean ± standard deviation. Significant differences were determined by ANOVA test; *p < 0.05.

b) Immunofluorescence images of MHC immunostaining (green) of differentiated myotubes after 6 days of culture and supplemented with different zinc concentrations.
